# A Multicenter Retrospective Chart Review Study of Treatment and Disease Patterns and Clinical Outcomes of Patients with Chronic-Phase Chronic Myeloid Leukemia in Third-Line Treatment or with T315I Mutation

**DOI:** 10.3390/cancers15164161

**Published:** 2023-08-18

**Authors:** Franck-Emmanuel Nicolini, Françoise Huguet, Lynn Huynh, Churong Xu, Christophe Bouvier, Aurore Yocolly, Gabriel Etienne

**Affiliations:** 1 Centre Léon Bérard, 69373 Lyon, France; 2 Fi-LMC Group, 69437 Lyon, France; huguet.francoise@iuct-oncopole.fr (F.H.); g.etienne@bordeaux.unicancer.fr (G.E.); 3 Hématologie, Institut Universitaire du Cancer de Toulose—Oncopole, 31100 Toulouse, France; 4 Analysis Group, Inc., Boston, MA 02199, USA; lynn.huynh@analysisgroup.com; 5 Analysis Group, Inc., Los Angeles, CA 90071, USA; 6 Novartis Services, Inc., East Hanover, NJ 07936, USA; 7 Institut Bergonié, 33076 Bordeaux, France

**Keywords:** chronic-phase chronic myeloid leukemia (CML-CP), tyrosine kinase inhibitor (TKI), treatment patterns, overall survival, T315I mutation

## Abstract

**Simple Summary:**

Many patients with chronic-phase chronic myeloid leukemia (CP-CML) treated with a tyrosine kinase inhibitor (TKI) experience disease progression and switch to another TKI, but each switch yields diminishing returns. To gain insight into the burden of repeated TKI treatment failures, this study analyzed the characteristics, treatment and disease patterns, and outcomes of adult patients with CP-CML in France whose disease progressed after treatment with two or more TKIs between 2006 and 2021. Patients switched TKIs up to six times; in many cases, treatment with the first and second TKIs lasted <2 years. On the other hand, patients who showed a good response to their third TKI had a lower risk of death. These findings provide a broad view of CP-CML treatment in France over the last 15 years and highlight the need for more effective therapies early in the treatment course that can improve outcomes for patients with CP-CML.

**Abstract:**

This retrospective chart review study investigated the clinical burden of adult patients with chronic-phase chronic myeloid leukemia (CP-CML) treated at three centers in France (2006–2021) who failed on two or more tyrosine kinase inhibitors (TKIs; third-line [3L]+ cohort) or harbored the *BCR::ABL1* T315I mutation (T315I cohort). In the 3L+ cohort (N = 157; median age at diagnosis, 56 years), TKIs received in 3L (median duration: 17 months) were dasatinib (32%), nilotinib (19%), imatinib (18%), ponatinib (17%), and bosutinib (14%). Of the 145 patients with documented responses in 3L, 42% experienced major molecular response (MMR) at 12 months. Median event-free survival [95% confidence interval] was 53.6 [44.0, 67.5] months, and median progression-free survival and overall survival (OS) were not reached. Achieving MMR in 3L was associated with a decreased mortality risk. In the T315I cohort (N = 17; 52 years), 41% of patients received five or more lines of therapy. Following identification of the T315I mutation, ponatinib was the most common TKI used (59%); the median [interquartile range] OS was 5 [3–10] years. The most common adverse events were infections (3L+ cohort) and thrombocytopenia (T315I cohort) (both 18%). Well-tolerated therapies that achieve durable responses are needed in 3L or earlier to improve CP-CML prognosis.

## 1. Introduction

Chronic myeloid leukemia (CML) is usually diagnosed in the chronic phase (CP) and can progress through the accelerated phase to blast crisis without effective treatment [1,2]. Although CML mortality has declined over the last two decades, the prevalence has increased [3]. The longer survival of patients and need for lifelong treatment has resulted in a greater disease burden on patients and society due to the negative impacts on quality of life (QoL) and adverse events (AEs) associated with traditionally used treatments.

Tyrosine kinase inhibitors (TKIs) are the frontline treatment for CML and have dramatically modified the disease course and clinical outcomes of patients [4]. The first TKI to be approved by the European Medicines Agency (EMA) was imatinib in November 2001; this was followed by the approval of the second-generation TKIs dasatinib in November 2006, nilotinib in November 2007, and bosutinib in March 2013, as well as the third-generation TKI ponatinib in July 2013 [5]. All are indicated for patients with previous TKI exposure, and imatinib, dasatinib, nilotinib, and bosutinib are also indicated in the frontline setting [6,7,8,9,10]. Despite the survival benefits conferred by TKIs, high rates of switching between TKIs—mainly due to intolerance or the development of resistance—have been reported in both clinical trials [11,12,13] and real-world studies [14,15,16,17,18]. In a retrospective study examining the treatment patterns of patients with CML in the United Kingdom (UK) between January 2013 and June 2018, 44% of patients had at least one TKI switch during the study period and 21% switched three or more times [17]; moreover, in an administrative database study conducted in Italy (January 2015–December 2018), treatment switching was observed in 26% of patients with three or more lines of treatment [14].

European LeukemiaNet (ELN) [19] and European Society for Medical Oncology [20] guidelines recommend that patients with CML who have failed ≥2 prior TKIs switch to an alternative second- or third-generation TKI in order to achieve an early and sustained molecular response (MR), which predicts longer progression-free survival (PFS) and overall survival (OS) [21]. However, the disease burden of CML has been shown to increase with each TKI treatment failure, as reflected by the greater use of medical services [22], with corresponding decreases in the probability of attaining an MR and long-term survival [16,23,24,25]. In particular, patients with the T315I mutation of the breakpoint cluster region–Abelson murine leukemia (*BCR::ABL1*) gene have a worse prognosis than those in the same phase of disease but lacking the mutation, and emergence of the T315I mutation in CP was shown to be associated with shorter OS, PFS, and failure-free survival [26].

To gain insight into the clinical burden associated with multi-TKI failure and the unmet therapeutic needs of patients with CML-CP, this study examined the characteristics, treatment patterns, and clinical outcomes of patients at three clinical centers in France who failed on at least two prior TKIs or who harbored the T315I mutation.

## 2. Materials and Methods

### 2.1. Study Design

This retrospective, multicenter chart review study was conducted at three large clinical reference institutions for CML in France (Centre Léon Bérard, Hématologie Institut Bergonié, and Institut Universitaire du Cancer Toulouse) and included patients with CML-CP who received three or more lines of therapy (3L+ cohort) or harbored the T315I mutation (T315I cohort) between 2006 and 2021. The patients received TKIs through international clinical trials or licensed or compassionate use. A schematic illustration of the study design is shown in Supplemental Figure S1. The index date for the 3L+ cohort was the date of initiation of 3L therapy. For the T315I cohort, the index date was defined as the date of treatment initiation after identification of the T315I mutation. The baseline (i.e., pre-index) period to describe patient clinical characteristics was defined as the 6 months preceding the index date, and the post-index follow-up period was the time from the index date to the date of last patient contact or patient death. Patients who were alive at the end of the follow-up period were censored at the date of last contact.

### 2.2. Study Population

Patients were eligible for inclusion in the study if they were aged ≥18 years at the time of CML-CP diagnosis. For the 3L+ cohort, additional inclusion criteria were the initiation of 3L therapy consisting of a TKI (bosutinib, dasatinib, imatinib, nilotinib, or ponatinib) or allogeneic stem cell transplantation (allo-SCT) after failing second-line therapy (2L). Additional inclusion criteria for the T315I cohort were the presence of the T315I mutation and receipt of TKI treatment or allo-SCT.

Exclusion criteria were as follows: history of other active malignancies within 3 years before CML-CP diagnosis; record of anticancer therapy for any other malignancies before initiation of 3L therapy or at the time of treatment initiation following identification of T315I mutation; and enrolment in a clinical trial at either of these time points.

### 2.3. Statistical Analysis

Descriptive statistics were used to summarize patient characteristics, treatment patterns/sequences, clinical outcomes, and AEs. Results are expressed as means (with standard deviations [SDs] and medians) or as frequencies (with proportions). For clinical outcomes, the cumulative incidence of patients achieving MR was summarized at specific time points. Event-free survival (EFS; where events included death, progression to accelerated phase or blast phase, treatment failure, and treatment discontinuation for any reason, whichever occurred first [19]), time to MR, time from 3L initiation to treatment discontinuation (TTD), PFS, and OS from 3L initiation in the 3L+ cohort were evaluated with the Kaplan–Meier (KM) method. Analyses of OS stratified by EUTOS long-term survival (ELTS) risk score and 2L resistance/intolerance status were also performed. An adjusted multivariate Cox proportional hazards model that included the following variables was used to identify prognostic factors impacting EFS and OS: age at index date, male sex, ELTS risk score at CML-CP diagnosis, number of comorbidities, additional chromosomal abnormalities at CML-CP diagnosis, achievement of major MR (MMR) in 3L, treatment with ponatinib, reason for terminating 2L was resistance or lack of efficacy, and reason for terminating 2L was intolerance or management of AEs.

## 3. Results

### 3.1. Patient Characteristics

The baseline characteristics of the 3L+ cohort (N = 157, 56.1% male) and T315I cohort (N = 17, 76.5% male) are shown in Table 1. The mean (SD) age at CML-CP diagnosis was 52.8 (15.7) and 51.1 (16.3) years, respectively. Comorbidities were reported in 137/157 patients in the 3L+ cohort (Supplemental Table S1). The mean (SD) number of comorbid conditions before the index date was 2.2 (1.8); the three most frequently observed comorbidities were cardiovascular diseases (54.8%), pulmonary disease/pulmonary arterial hypertension (21.7%), and gastrointestinal issues (13.4%). Among the 89 patients (56.7%) assessed for *BCR::ABL1* genetic mutations at 3L initiation, the most common mutations found were T315I (7.9%), F359I (3.4%), and M244V (3.4%). The T315I cohort included the seven aforementioned patients in the 3L+ cohort with the T315I mutation; most patients in this cohort (41.2%) were classified as high risk at CML-CP diagnosis based on the ELTS risk score.

### 3.2. Treatment Patterns/Sequences

**Figure 1:**
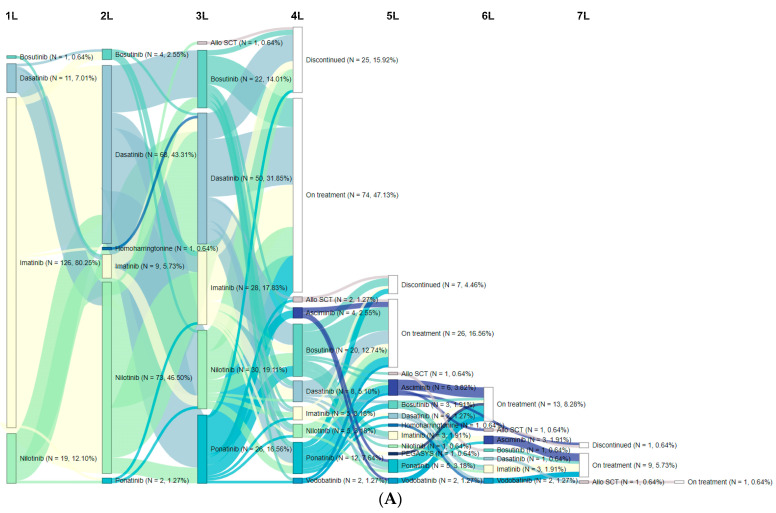

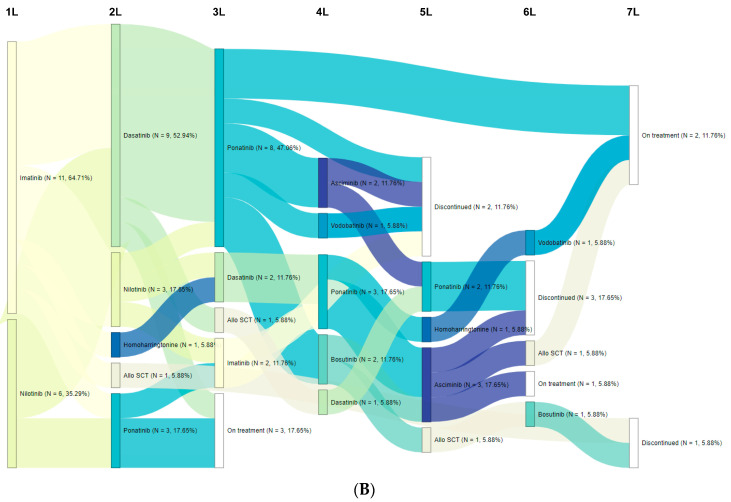
Sankey diagram of treatment patterns for CML-CP patients: (**A**) Patients with three or more lines of therapy. (**B**) Patients with the T315I mutation. Abbreviations: 1/2/3/4/5/6/7L, first/second/third/fourth/fifth/sixth/seventh line; Allo-SCT, allogeneic stem cell transplantation.

Patients in the 3L+ cohort had, on average, 3.6 (0.9) lines of therapy; 36.9% had ≥4 lines and 15.9% had ≥5 lines (Figure 1A). Patients were on 3L therapy for a median time of 17.0 months; the median (95% confidence interval [CI]) TTD in the KM analysis was 55.0 (46.7, 74.6) months (Supplemental Table S2). The most common treatment received in 3L was dasatinib (31.8%), followed by nilotinib (19.1%), imatinib (17.8%), ponatinib (16.6%), bosutinib (14.0%), and allo-SCT (0.6%) (Figure 1A). The most frequent treatment sequence starting from the first line (1L) was imatinib→nilotinib→dasatinib (17.2%) and imatinib→dasatinib→nilotinib (9.6%). Dasatinib was the most frequently used TKI in the last line of therapy (23.6%). Approximately 50% of patients discontinued treatment; the most common reasons for 3L discontinuation were AEs or intolerance (54/78 [69.2%]), resistance (18/78 [23.1%]), and signs of ineffectiveness (14/78 [17.9%]).

The T315I mutation was identified in 2L (n = 6, 35.3%), 3L (n = 5, 29.4%), 4L (n = 4, 23.5%), and 5L (n = 2, 11.8%). Patients with the T315I mutation had, on average, 3.9 (1.5) lines of therapy; 52.9% had ≥4 lines and 41.2% had ≥5 lines (Figure 1B). The mean (SD) duration of the line of therapy identified as the T315I line of interest for patients in the T315I cohort was 18.5 (20.6) months (Supplemental Table S3). Treatments received by these patients were ponatinib (58.8%), dasatinib (17.6%), asciminib (11.8%), and allo-SCT (11.8%) (Figure 1B). The most common treatment sequences were imatinib→dasatinib→ponatinib (17.6%), and nilotinib→ponatinib (11.8%). In the last line of therapy, the most common treatments were ponatinib (n = 7, 41%) and asciminib (n = 3, 18%) through compassionate use or clinical trials. Nearly two-thirds of patients (64.7%) discontinued treatment; the most common reasons for discontinuation were AEs or intolerance (9/11 [81.8%]), resistance (4/11 [36.4%]), and signs of ineffectiveness (2/11 [18.2%]).

### 3.3. Clinical Outcomes

#### 3.3.1. Molecular Response

**Figure 2:**
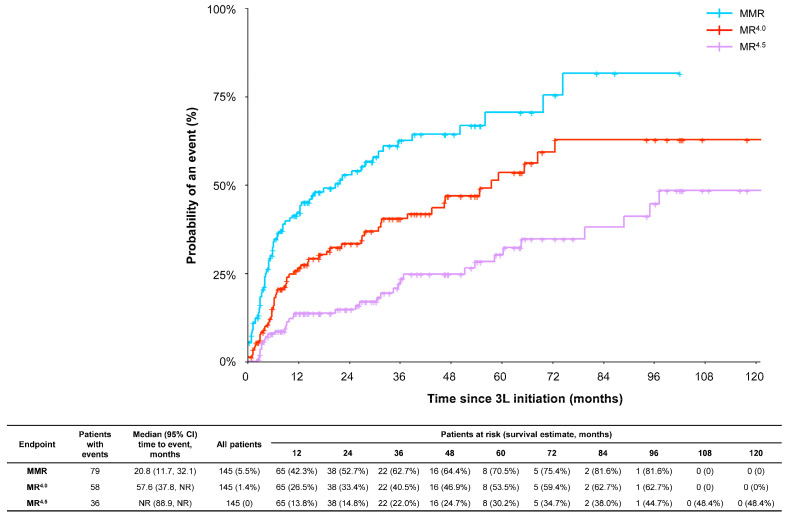
Cumulative incidences of molecular responses in patients with CML-CP with three or more lines of therapy. MMR, 0.01% < *BCR::ABL1* ≤ 0.1% or 3-log reduction; MR^4.0^, 0.0032% < *BCR::ABL1* ≤ 0.01% or 4-log reduction; and MR^4.5^, 0.001% ≤ *BCR::ABL1* ≤ 0.0032% or 4.5-log reduction. Abbreviations: CI, confidence interval; MMR, major molecular response; MR, molecular response; NR, not reached.

During 3L therapy, 54.8% of patients (n = 86) showed a complete cytogenetic response (CCyR; defined as an absence of Philadelphia chromosome-positive metaphases as measured by bone marrow cytogenetics). Of the patients with documented responses in 3L (N = 145), over half (54.5%) achieved MMR in 3L in a median time of 20.8 months; over one-third (40%) achieved MR^4.0^ in a median time of 57.6 months, and 24.8% achieved MR^4.5^ with median time to event not reached (Figure 2). The rate of MMR, MR^4.0^, and MR^4.5^ at 12 months was 42%, 26%, and 14%, respectively. Sustained MR^4.0^ (defined as achieving MR^4.0^ or better [i.e., *BCR::ABL1* ≤ 0.01%] in all consecutive assessments performed for at least 12 months [i.e., 365.25 days]) was achieved by 33.1% of patients for a median duration of 87.1 months, and sustained MR^4.5^ (defined as achieving MR^4.5^ or better [i.e., *BCR::ABL1* ≤ 0.0032%] in all consecutive assessments performed for at least 2 years [i.e., 730.5 days]) was achieved by 11.7% of patients for a median duration of 134.2 months. Treatment-free remission (TFR) was observed in 16 patients (10.2%) in 3L (median duration: 45.3 months). Among the 16 patients in TFR, five (31.3%) were intolerant to 3L TKI.

Among patients with T315I mutation, seven achieved MMR (three patients in 2L and four in 3L), three achieved MR^4.0^ (one patient in 2L and two in 3L), and none achieved MR^4.5^. No patient in this cohort initiated TFR.

#### 3.3.2. Survival

**Figure 3:**
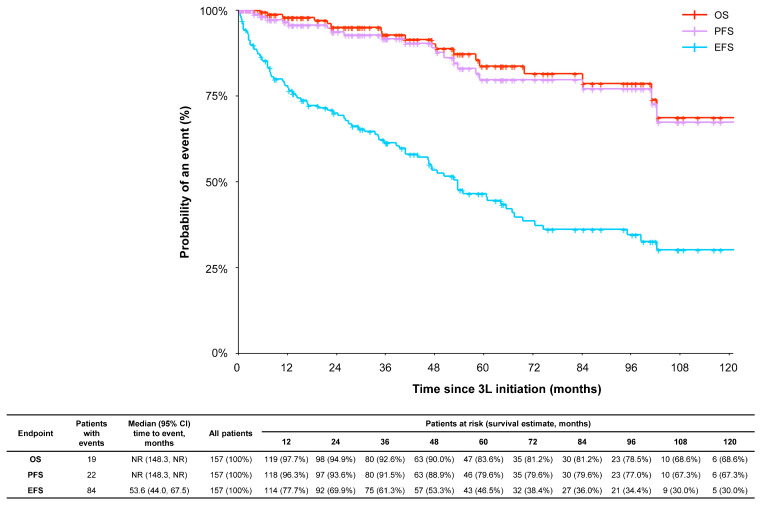
Survival outcomes, including EFS, PFS, and OS in patients with CML-CP with three or more lines of therapy. An event was defined per European LeukemiaNet as death, progression to accelerated phase or blast phase, treatment failure, and treatment discontinuation for any reason, whichever occurred first [19]. Abbreviations: 3L, third line; CI, confidence interval; EFS, event-free survival; OS, overall survival; PFS, progression-free survival; NR, not reached.

The survival outcomes of patients in the 3L+ cohort are shown in Figure 3. The overall median EFS was 53.6 months (95% CI: 44.0, 67.5 months). Overall median PFS and OS were not reached as of data collection for all patients in the 3L+ cohort; 19 patients (12.1%) had died as of data abstraction due to disease progression (n = 7 [36.8%]), toxicity (n = 1 [5.3%]), or other reasons (n = 10 [52.6%]) including bacterial infection, cardiac complications of renal failure, cardiac insufficiency, congestive heart failure, diabetes, general impairment, sepsis, stroke, and unclassified infection. The cause of death was unknown in one patient. In a subgroup analysis of patients in the 3L+ cohort with intermediate ELTS risk (vs. patients with high, low, and not assessed/unknown risk), the median OS (95% CI) was 102.4 (102.4, not reached) months.

Factors significantly impacting EFS and OS in the 3L+ cohort were evaluated in a multivariate Cox regression model adjusted for key demographic and clinically relevant characteristics including age at index date, sex, ELTS risk score at CP-CML diagnosis, number of comorbidities, additional chromosomal abnormalities at CP-CML diagnosis, achievement of MMR in 3L, receipt of ponatinib, lack of efficacy as the reason for terminating 2L therapy, and intolerance or management of AEs as the reason for terminating 2L therapy (Table 2). For each additional year of age at the index date, patients had a 2% higher risk of an event (i.e., disease progression; hazard ratio [HR] = 1.02, 95% CI: 1.00, 1.04) and 7% higher risk of death (HR = 1.07, 95% CI: 1.01, 1.13) after adjusting for all other covariates (both *p* < 0.05). In terms of clinically relevant characteristics, patients with additional chromosomal abnormalities at CP-CML diagnosis had a 135% higher risk of an event (adjusted HR = 2.35, 95% CI: 1.27, 4.34) and 602% higher risk of death (adjusted HR = 6.02, 95% CI: 1.78, 20.36) compared with patients without abnormalities (both *p* < 0.01). On the other hand, patients who achieved MMR in 3L had a 78% lower risk of an event (adjusted HR = 0.22, 95% CI: 0.13, 0.37; *p* < 0.001) and 90% lower risk of death (adjusted HR = 0.10, 95% CI: 0.02, 0.51; *p* < 0.01) than those not achieving MMR in 3L. There was no significant association between the other variables and EFS or OS.

Among patients with the T315I mutation, the median (range) OS since T315I identification was 5 (3–10) years; three patients (17.6%) had died as of data abstraction due to disease progression (n = 1 [33.3%]) or stroke-related reasons (n = 2 [66.7%]).

### 3.4. AEs in 3L

AEs were documented in 139/157 patients (89%) in the 3L+ cohort and 14/17 patients (82.4%) in the T315I cohort (Supplemental Table S4). Patients had, on average, 2.7 AEs during 3L therapy. The most common AEs were infections (17.8%), asthenia (13.4%), and abdominal pain (12.7%). Among patients with T315I mutation, thrombocytopenia (18%) was the most common AE.

## 4. Discussion

The introduction of TKIs targeting the tyrosine kinase activity of the BCR::ABL oncoprotein has transformed the management of CP-CML [27], increasing relative survival rates to a point where CML is no longer considered an incurable disease. Imatinib is the most frequently prescribed 1L TKI in France [28]; however, over half of patients with CP-CML develop resistance or intolerance to imatinib, and up to 40% who receive second-generation TKIs (dasatinib, nilotinib, or bosutinib) fail to show a deep MR (MR^4.5^) after 5 years [29,30,31]. Moreover, CP-CML patients harboring the T315I mutation are resistant to first- and second-generation TKIs [30]. To gain insight into the disease burden of these patient populations in France, this retrospective multicenter chart review study examined the characteristics, treatment patterns, and outcomes of real-world CP-CML patients who failed multiple lines of treatment with TKIs or who harbored the T315I mutation. Consistent with previous reports [11,12,13,14,15,16,17,18], we found that a large proportion of patients switched to another treatment (after a median time of 17 and 11 months in the 3L+ and T315I cohorts, respectively) or discontinued treatment altogether (50% and 65%, respectively), mainly due to intolerance, resistance, and signs of ineffectiveness. Additionally, less than half of patients with documented responses had MR^4.0^ or MR^4.5^, and most patients experienced AEs. These results indicate that the therapeutic needs of patients with CP-CML in France are not being met by currently available TKIs.

The standard of care beyond 2L therapy is not well defined by CML treatment guidelines. ELN 2020 recommends any TKI that was not used in 1L or 2L for patients developing resistance and/or intolerance, with ponatinib being favored over second-generation TKIs for eligible patients, especially those with T315I mutation [20]. In the present study, dasatinib was the most frequently used TKI in 3L. It should be noted that nearly half of the patients (45%) in this study initiated 3L before bosutinib and ponatinib were eligible for reimbursement in France (February 2015 and June 2016, respectively). The rates observed in this study differ slightly from those reported in a noninterventional, descriptive cohort study based on CML registries in Czechia, the Netherlands, and Sweden [32]. This is expected given the different approval dates of TKIs across countries; in these patients, nilotinib was the most frequently prescribed TKI in third or later lines of therapy (45–60%), followed by dasatinib (17–38%), bosutinib (0–28%), and ponatinib (11–17%). The clinical benefits of TKI treatment have been shown to diminish with successive lines of therapy: for example, response rates to ponatinib were higher among CP-CML patients who had received fewer prior TKIs [33]. Although median PFS and OS were not reached in the 3L+ cohort after a mean follow-up of 66.9 months from the index date, among the 103 patients in this cohort who were tested for cytogenetic response, only 55% showed CCyR during 3L therapy; moreover, among the 145 patients who were tested for MR, only half had an MMR after a median time of 20.8 months, and less than one-quarter showed MR4.5. A shorter time to MMR with TKI treatment has been shown to predict a higher complete MR rate in CML patients [34], and MMR and CCyR have been suggested as surrogate endpoints for survival in studies of CML patients [35,36]. On the other hand, failure of 3L TKI therapy is associated with low response rates in subsequent lines of treatment and higher rates of disease progression and mortality [23,37]; among patients with CP-CML in the UK who failed 1L imatinib and 2L dasatinib or nilotinib, 35% showed a CCyR and 19% had MMR [24], with CyR to 1L imatinib or a 2L therapy being independent predictors of CCyR in 3L. Thus, improving response rates early in the treatment course may achieve better long-term outcomes.

As patients with CML live longer, an increasingly important aspect of patient care is QoL [38], which includes minimizing or adequately managing AEs. Although deep MRs are achieved more rapidly with second- and third-generation TKIs than with imatinib, the therapeutic benefit is accompanied by greater toxicity [5], which could influence treatment choices. Only half of the patients in the 3L+ cohort remained on 3L therapy at the time of data collection; in the majority of cases, patients discontinued 3L therapy because of AEs/intolerance (69%) and the development of resistance (23%), with an average of 2.7 AEs reported and most patients (89%) experiencing at least one AE during 3L therapy. Of particular concern for CML patients with comorbidities are cardiovascular AEs, which have been frequently reported in clinical trials and real-world studies of later-generation TKIs [39,40] and greatly limit the therapeutic options for patients with cardiovascular risk factors [30]. Patients in the 3L+ cohort had, on average, 2.2 comorbid conditions at the index date, with cardiovascular diseases being the most common (55%); moreover, 44% of patients had a cardiovascular risk factor, mainly hypertension and hyperlipidemia/dyslipidemia. Therefore, alternative treatments that are safe for patients with cardiovascular disease or risk factors—as well as for patients who have failed existing therapies—are needed. Several novel therapies including third-generation (olverembatinib [41,42] and vodobatinib [43,44]) and fourth-generation [45,46] TKIs are currently under investigation for third- or later-line treatment of CML, with some being evaluated for their efficacy in patients with the T315I mutation. Asciminib, the first BCR::ABL inhibitor targeting the myristoyl pocket rather than the ATP-binding site of the ABL kinase domain, was approved by the European Commission on 25 August 2022, for the treatment of patients with CP-CML after failure on two or more TKIs [47,48,49] (with approval not yet sought for patients with the T315I mutation). The efficacy and tolerability of asciminib in patients with CML-CP with multi-TKI failure have also been demonstrated in real-world studies conducted in various countries, where it has been available for compassionate use since 2016 [50,51,52,53,54].

The emergence of new mutations including T315I that reduce sensitivity to alternative TKIs after failure on imatinib has been reported during sequential TKI treatment [55]. The T315I mutation is associated with resistance to first- and second-generation TKIs [30] and poor survival outcomes [26,56]. The frequency of this mutation in CML patients who failed on imatinib (with or without previous interferon-α treatment) was found to range between 10% and 27% [26] and was 19% in a previous multicenter study conducted in France [57]. These rates are higher than what was observed in our study: of the 89 (57%) patients assessed for *BCR::ABL1* mutations prior to 3L initiation, 8% had the T315I mutation. Just one patient with this mutation initiated a subsequent line of therapy before 2010. This low number may be explained by the fact that, in the 2009 ELN recommendations for CML management, allo-SCT was the only treatment option for patients who failed on imatinib [58]. In subsequent years, the proportion of patients initiating later lines of therapy increased markedly to 53% between 2010 and 2014 and to 41% between 2015 and 2019, possibly reflecting the 2013 approval of ponatinib by the EMA as the only TKI effective against the T315I mutation and its recommendation in 2013 ELN guidelines [19]. However, less than half of the patients in the T315I cohort (~40%) achieved MMR, which was similar to the proportion of patients in the 3L+ cohort. This suggests that, regardless of the TKI and lines of therapy received, patients who develop the T315I mutation have as much need for more effective treatment options as those who fail on three or more TKIs.

This study had certain limitations that should be noted. As with all retrospective nonrandomized studies, the analyses may have been affected by uncontrolled confounding; reporting, selection, or recall bias; or nonrandom missing data. The occurrence of positive selection bias was limited by the inclusion of data from eligible patients (currently living or deceased) up until the date of last contact or date of death. Additionally, to mitigate potential inconsistencies in treatment responses among subjects across the three clinical centers, the responses were recorded using the IS. Any data that were not recorded in patient charts were not included in the database for analysis; consequently, data on comorbidities and AEs may have been missing for some patients. Finally, the three clinical sites included in the study may not be representative of the 75 clinical sites in France or those in other countries with different reimbursement or practice patterns, which could limit the generalizability of the findings.

## 5. Conclusions

The results of this study provide a broad perspective of CP-CML management in France over the last 15 years. During this period, treatment patterns have evolved according to the changing therapeutic landscape for CML as well as physicians’ experiences in treating patients in real-world practice settings. CP-CML patients at three medical centers in France who were treated with three or more lines of therapy received up to seven lines of therapy, and those harboring the T315I mutation received up to six lines. Earlier lines of treatment lasted less than 2 years in CML-CP patients in 3L and less than 1 year in those harboring the T315I mutation, indicating an enduring disease burden and poor prognosis in these patients with traditional TKIs. Separately, this study found that, after adjusting for key clinical variables, achieving MMR in 3L was associated with a lower risk of mortality, which can serve as an important indicator for long-term clinical benefits. These findings underscore the need for novel therapies in 3L or earlier in the treatment course that are well tolerated and can achieve durable responses to improve the prognosis of patients with CP-CML.

## Figures and Tables

**Table 1:** Baseline characteristics of the study population.

Characteristic	3L+ Cohort N = 157	T315I Cohort N = 17
Medical center		
Centre Léon Bérard, Lyon	65 (41.4)	8 (47.1)
Hématologie Institut Bergonié, Bordeaux	61 (38.9)	5 (29.4)
Institut Universitaire du Cancer Toulouse, Toulouse	31 (19.7)	4 (23.5)
Sex		
Male	88 (56.1)	13 (76.5)
Female	69 (43.9)	4 (23.5)
Age at CML-CP diagnosis, years	52.8 ± 15.7 [55.7]	51.1 ± 16.3 [52.2]
ELTS risk score at CML-CP diagnosis		
Low risk (≤1.5680)	63 (40.1)	3 (17.6)
Intermediate risk (>1.5680 to ≤2.2185)	39 (24.8)	4 (23.5)
High risk (>2.2185)	17 (10.8)	7 (41.2)
Not assessed	2 (1.3)	−
Unknown/not sure	36 (22.9)	3 (17.6)
Year of CML-CP diagnosis		
Before 2010	88 (56.1)	10 (58.8)
On or after 2010	69 (43.9)	7 (41.2)
Time from CML-CP diagnosis to index date, months	78.6 ± 56.8 [61.6]	66.9 ± 70.6 [51.1]
Age at index date ^1^, years	59.3 ± 15.6 [62.1]	56.3 ± 13.1 [55.0]
Length of follow-up ^2^, months	66.9 ± 43.3 [59.3]	68.3 ± 48.2 [55.2]
*BCR::ABL1* rearrangement at CML-CP diagnosis		
Major	142 (90.4)	15 (88.2)
Minor	4 (2.5)	1 (5.9)
Other	11 (7.0)	1 (5.9)
*BCR::ABL1* mutation status		
Not assessed	67 (42.7)	−
Unknown/not sure	1 (0.6)	−
Assessed	89 (56.7)	−
T315I	7 (7.9)	−
Year T315I mutation was detected		
Before 2015	−	11 (64.7)
On or after 2015	−	6 (35.3)
Line of therapy in which T315I mutation was identified		
2L	−	6 (35.3)
3L	−	5 (29.4)
4L	−	4 (23.5)
5L	−	2 (11.8)
Additional chromosomal abnormalities at CML-CP diagnosis		
Yes	24 (15.3)	6 (35.3)
No	133 (84.7)	11 (64.7)

Data are shown as mean ± standard deviation [median] or n (%). Abbreviations: 3L+, three or more lines of therapy; *BCR::ABL1*, breakpoint cluster region–Abelson murine leukemia 1 gene fusion; CML-CP, chronic myeloid leukemia in chronic phase; ELTS, EUTOS long-term survival. Notes: ^1^ The index date was defined as the date of initiation of third-line therapy for the 3L+ cohort, and the date of initiation of tyrosine kinase inhibitor or allogeneic stem cell transplantation after identification of T315I mutational status for the T315I cohort. ^2^ Length of follow-up was defined as the time from the index date to the date of last known contact with patient or patient death.

**Table 2:** Multivariate Cox regression models of EFS and OS in patients receiving three or more lines of therapy.

	EFS			OS		
	HR	95% CI	*p* Value	HR	95% CI	*p* Value
Multivariate model (N = 145 ^1^)						
Age (years) at index date	1.02	(1.00, 1.04)	<0.05 *	1.07	(1.01, 1.13)	<0.05 *
Male (ref: Female)	0.89	(0.54, 1.47)	0.65	0.81	(0.24, 2.70)	0.73
ELTS risk score at CML-CP diagnosis (ref: Low risk)						
Intermediate risk	0.62	(0.31, 1.21)	0.16	0.81	(0.11, 5.73)	0.83
High risk	1.10	(0.50, 2.43)	0.81	2.76	(0.30, 25.00)	0.37
Not assessed or unknown	0.80	(0.43, 1.48)	0.47	2.17	(0.45, 10.38)	0.33
Number of comorbid conditions	1.13	(0.97, 1.31)	0.12	1.12	(0.81, 1.55)	0.48
Additional chromosomal abnormalities at CML-CP diagnosis (ref: No additional abnormalities)	2.35	(1.27, 4.34)	<0.01 *	6.02	(1.78, 20.36)	<0.01 *
MMR was achieved in 3L (ref: MMR was not achieved in 3L)	0.22	(0.13, 0.37)	<0.001 *	0.10	(0.02, 0.51)	<0.01 *
Treatment with ponatinib (ref: Non-ponatinib)	0.96	(0.45, 2.02)	0.91	1.36	(0.18, 10.28)	0.76
Reason for terminating 2L is resistance or lack of efficacy (ref: No)	1.51	(0.83, 2.73)	0.18	1.58	(0.32, 7.78)	0.57
Reason for terminating 2L is intolerance or management of AEs (ref: No)	0.64	(0.34, 1.18)	0.15	0.80	(0.15, 4.22)	0.79

* Statistically significant at *p* < 0.05. Abbreviations: 2/3L, second/third line; AE, adverse event; CI, confidence interval; CML-CP, chronic myeloid leukemia in chronic phase; EFS, event-free survival; ELTS, EUTOS long-term survival; HR, hazard ratio; MMR, major molecular response; OS, overall survival; ref, reference category. Note: ^1^ 12 Patients were not tested for molecular response in 3L and were therefore not included in the analysis.

## Data Availability

The datasets generated and analyzed during the current study are not publicly available because they were used pursuant to a data use agreement.

## Supplementary Materials

The following supporting information can be downloaded at: https://www.mdpi.com/article/10.3390/cancers15164161/s1, Table S1. Comorbidities in patients with CML-CP with three or more lines of therapy, Table S2. Treatment patterns of patients with chronic myeloid leukemia who received third-line therapy, Table S3. Treatment patterns of patients with chronic myeloid leukemia with T315I mutation, Table S4. Adverse events during third-line treatment for chronic myeloid leukemia, Figure S1. Study design scheme.
