# Supplementary material for: A Multicenter Retrospective Chart Review Study of Treatment and Disease Patterns and Clinical Outcomes of Patients with Chronic-Phase Chronic Myeloid Leukemia in Third-Line Treatment or with T315I Mutation
Source: Cancers (Basel). 2023 Aug 18;15(16):4161. doi: 10.3390/cancers15164161 (PMC10453285; doi:10.3390/cancers15164161)

SUPPLEMENTAL TABLES AND FIGURE

Supplemental Table S1. Comorbidities in patients with CML-CP with three or more lines of therapy

	**3L+ cohort N = 157**
Number of comorbid conditions[1]	2.2 ± 1.8 [2.0]
Comorbid conditions, n (%)	
Cardiovascular disease[2]	86 (54.8)
Pulmonary disease/pulmonary arterial hypertension	34 (21.7)
Gastrointestinal issues[3]	21 (13.4)
Renal disease[4]	18 (11.5)
Diabetes	16 (10.2)
Liver disease[5]	9 (5.7)
Other[6]	99 (63.1)
No comorbidities	19 (12.1)
Unknown / not sure	1 (0.6)
Any ischemic condition, n (%)	88 (56.1)
Cardiovascular disease[2]	86 (97.7)
Cerebrovascular disease[7]	9 (10.2)
Cardiovascular risk factors, n (%)	69 (43.9)
Hypertension	43 (62.3)
Hyperlipidemia/dyslipidemia	33 (47.8)
Diabetes	16 (23.2)
Obesity	8 (11.6)

Data are shown as mean ± standard deviation [median] or n (%).

Abbreviations: 3L+, three or more lines of therapy; CML-CP: chronic myeloid leukemia in chronic phase.

Notes:

[1] Number of comorbid conditions accounted for all comorbid conditions (all types of cardiovascular disease, cerebrovascular disease, connective tissue disease, chronic obstructive pulmonary disease, dementia, diabetes, liver disease, moderate or severe renal disease, non-hematologic cancer, obesity, or peptic ulcer disease) that patients experienced up until 3L.

[2] Cardiovascular diseases included aortic aneurysm, aortic insufficiency, arrhythmias, atrial dilation, cardiac decompensation, cardiac insufficiency, cardiomyopathy, chronic cardiomyopathy, coarctation of the aorta, congestive heart failure, coronary artery bypass graft, coronary artery disease, coronary insufficiency, decompensation of a myocardiopathy, dilated cardiomyopathy, dyslipidemia, heart murmur, hypertension, hypertrophic cardiopathy, ischemic cardiac failure, ischemic cardiomyopathy, little ejection systolic aortic murmur, myocardial infarction, myocardiopathy, pacemaker, percutaneous coronary intervention, pericarditis, peripheral vascular disease, systolic aortic murmur, systolic murmur, triple room cardiac defibrillator, or unstable angina.

[3] Gastrointestinal issues included bloating, colic perforation, constipation, Crohn disease, diarrhea, digestive troubles, dyspepsia, frequent vomiting, gastroesophageal reflux, hemorrhoids, irritable bowel syndrome, nausea, persistent digestive intolerance, or right hemicolectomy.

[4] Renal diseases included bladder lithiasis operated, bladder polyps, chronic kidney disease, hemodialysis, history of renal transplant, hyperuricemia, multiple kidney lithiasis, nephritis or nephropathy, peritoneal dialysis, renal colic, renal osteodystrophy, sequelae of paraplegia (bladder), uricemia, uricemia treated, or urinary infections.

[5] Liver diseases included chronic hepatitis, esophageal varices, hepatic coma, hepatorenal syndrome, hepatosplenomegaly, other sequelae of chronic liver disease, or portal hypertension.

[6] Other comorbid conditions included actinic keratosis, addiction, age-related macular degeneration, alcoholism, algodystrophy, allergy, alopecia, amyotrophic lateral sclerosis, anal margin abscess, anemia, ankylosing spondylitis, anorexia, anxiety, anxiodepressive syndrome, arthrosis, arthritis, arthromyalgia, articular pain, asthenia, autograft, axillary dissection, benign follicular lymph node hyperplasia, bilateral gonarthrosis, bilateral hearing loss, bilateral medullary necrosis of both ankles, bilateral ovariectomy, bilateral salpingoophorectomy, bilateral stripping, bipolar disorders, bone pain, both hips prothesis, bullous pemphigoid, carotid atheroma, carotid stenosis, cataract, cerebral malaria, cerebral oedema, cervical disc herniation, cervical odontoid fracture, cervical pain, cervicalgia, cholecystectomy, cholinergic urticaria, chronic asthenia, chronic lumbosciatica, cognitive disorders, complete excision of a basal cell carcinoma, conjunctivitis, contact dermatitis, cortical blindness, cramps, degenerative neuropathy of the lower limbs associated with arthralgia, depressive syndrome, diffuse large B-cell lymphoma, drepanocytosis, drug eruption, dry eye syndrome, Dupuytren disease, dysarthria, dyspnea, eczema urticaria, oedema, epigastralgia, erection disorder, essential epilepsia, exposed to handling solvents, flutter removal, general condition alteration, genital candidiasis, giant urticaria in childhood, glaucoma, gout, Guillain Barré syndrome, gynecomastia, hematologic toxicity, Hashimoto's thyroiditis, hay fever, headache, hearing aid, hepatitis B, herniated disc operated, herpes, HIV+, hypercholesterolemia, hyperleukocytosis, hyperparathyroidism, hyperuricemia, hypodermatitis, hypogammaglobulinemia, hypokalemia, hypothyroidism, hysterectomy, iliac artery angioplasty, inguinal hernia operated, insomnia, internal carotid stenosis, intraductal papillary mucinous neoplasm, knee gout crisis, hypercholesterolemia, labial herpes, left breast tumorectomy, left hypochondrium pain without splenomegaly, lombalgias, low back pain, lower limbs arthromyalgias, lumbar pain, lupus, mastocytosis, Ménière's disease, metabolic syndrome, monoclonal dysglobulinemia of undetermined significance, mood disorders, multiple kidney lithiasis, musculoskeletal pain, myelodysplastic syndrome, nasal polyposis, night sweats, oedemas, oligoarthritis, optic neuritis, oral mycosis, osteopenia, ovariopexy, palpebral oedema, pancytopenia, papular erythroderma, paraplegia, pelade, peripheral neuropathy, persistent cervicalgia, polyarthritis, polydipsia, polyuria, presence of herpes in the throat, primary hyperaldosteronism, professional toxic exposure to glues and solvents, prostate adenoma, prostate resection, prostatic adenocarcinoma treated by curietherapy, pruritus, psoriasis, mental disorders, psychiatric problems, psychological problems, pyrosis, Raynaud's syndrome, recurrent anal margin abscess, recurrent ear infection, recurrent nose infection, recurrent throat infection, rheumatoid arthritis, right breast tumorectomy, right cataract operated, right tinnitus, salpingo-oophorectomy, scalp psoriasis, sciatica, seborrheic dermatitis, sensory and motor neuropathy, sensory neuropathy, seronegative rheumatoid arthritis, severe anxiety disorders, sexual impotence, shoulders pain, skin lesions, skin troubles, skin whitening, sleep apnea syndrome, solar keratosis, spasmophilia, spinal osteoarthritis, stent, stiff persons syndrome, thrombocytopenia, thrombocytosis, thrombopenia, thrombosis, thyroidectomy, tinea versicolor, tinnitus, toe gout, tonsillectomy, total hysterectomy, total thyroidectomy, type 2 diabetes, varnishes, vascular algia, Verneuil's disease, vertebral hyperostosis, vesical lithiasis, vitiligo, or xanthelasma.

[7] Cerebrovascular diseases included cerebral oedema, cortical hematoma, epilepsy, transient ischemic attack, subdural hematoma, vascular cerebral attack, cerebrovascular diseases with mild or no residual transient ischemic attack, or cerebrovascular diseases with neurologic deficit including hemiplegia.

Supplemental Table S2. Treatment patterns of patients with chronic myeloid leukemia who received third-line therapy

		Reason for terminating 2L[1]	
	3L<br>N = 157	Resistance/lack of efficacy<br>N = 49	Intolerance or management of adverse events<br>N = 109
Year patient initiated 3L			
Before 2010	14 (8.9)	7 (14.3)	10 (9.2)
2010–2014	57 (36.3)	13 (26.5)	39 (35.8)
2015–2019	68 (43.3)	27 (55.1)	45 (41.3)
2020–2021	18 (11.5)	2 (4.1)	15 (13.8)
Duration of 3L, months	26.9 ± 25.3 [17.0]	31.1 ± 26.6 [22.8]	25.0 ± 25.1 [16.9]
Time from 2L discontinuation to 3L initiation, months	2.5 ± 6.0 [0.3]	0.7 ± 1.4 [0.0]	2.4 ± 6.0 [0.5]
Type of therapy received in 3L			
Dasatinib	50 (31.8)	13 (26.5)	32 (29.4)
Duration, months	30.4 ± 28.5 [25.2]	46.9 ± 30.5 [52.8]	30.0 ± 28.3 [25.4]
Nilotinib	30 (19.1)	7 (14.3)	24 (22.0)
Duration, months	36.1 ± 28.0 [39.4]	38.3 ± 23.8 [35.7]	35.1 ± 30.6 [46.5]
Imatinib	28 (17.8)	2 (4.1)	24 (22.0)
Duration, months	17.6 ± 13.8 [12.8]	9.4 ± 2.9 [9.4]	16.7 ± 14.7 [10.7]
Ponatinib	26 (16.6)	22 (44.9)	9 (8.3)
Duration, months	23.2 ± 23.6 [12.8]	20.4 ± 22.6 [12.3]	19.0 ± 19.3 [12.4]
Bosutinib	22 (14.0)	4 (8.2)	20 (18.3)
Duration, months	16.5 ± 16.7 [8.6]	25.0 ± 20.2 [19.6]	13.1 ± 14.7 [8.1]
Allo-SCT	1 (0.6)	1 (2.0)	0 (0.0)
Patients still on 3L therapy as of data collection date	79 (50.3)	19 (38.8)	56 (51.4)
Median (95% CI) time to discontinuation of 3L therapy, months	55.0 (46.7, 74.6)	–	–
Patients discontinued 3L	78 (49.7)	30 (61.2)	53 (48.6)
Reasons for 3L discontinuation[1]			
Adverse events or intolerance[2,3]	54 (69.2)	20 (66.7)	42 (79.2)
Resistance[4]	18 (23.1)	15 (50.0)	9 (17.0)
Signs of ineffectiveness[5]	14 (17.9)	11 (36.7)	9 (17.0)
Failure to achieve response	5 (6.4)	3 (10.0)	4 (7.5)
Progression to accelerated phase or blast phase	5 (6.4)	5 (16.7)	2 (3.8)
Suboptimal response	3 (3.8)	2 (6.7)	3 (5.7)
Loss of response	2 (2.6)	1 (3.3)	1 (1.9)
Other reasons	23 (29.5)	8 (26.7)	14 (26.4)
Initiation of treatment-free remission corresponding to treatment discontinuation	16 (20.5)	2 (6.7)	11 (20.8)

	3L	Reason for terminating 2L[1]	
		Resistance/lack of efficacy	Intolerance or management of adverse events
	N = 157	N = 49	N = 109
Acquired mutations[6]	6 (7.7)	5 (16.7)	3 (5.7)
Convenience	1 (1.3)	1 (3.3)	0 (0.0)
Pregnancy	0 (0.0)	0 (0.0)	0 (0.0)
Vacation	0 (0.0)	0 (0.0)	0 (0.0)
Economic considerations	0 (0.0)	0 (0.0)	0 (0.0)
Non-adherence	0 (0.0)	0 (0.0)	0 (0.0)
Patient preference	0 (0.0)	0 (0.0)	0 (0.0)
Unknown / not sure	0 (0.0)	0 (0.0)	0 (0.0)

Data are shown as mean ± standard deviation [median] or n (%) unless otherwise indicated.

Abbreviations: 2/3L, second/third line; Allo-SCT, allogeneic stem cell transplantation; SD, standard deviation.

Notes:

[1] Patients may have had more than one reason for treatment switch between lines of therapy; therefore, the sum may exceed 100%.

[2] Included adverse events and drug–drug interactions.

[3] Intolerance (i.e., drug–drug interactions) was specified as determined by the physician.

[4] Resistance was specified as determined by the physician.

[5] Signs of ineffectiveness was defined as failure to achieve response, suboptimal response, loss of response, or progression to accelerated phase or blast phase.

[6] Acquired mutations included A380G, E255K, E255V, E355G, F317L, F359C, F359I, F359V, H396R, L248L, M244V, M351T, Q252H, V225I, V299L, V379I, and Y253H.

Supplemental Table S3. Treatment patterns of patients with chronic myeloid leukemia with T315I mutation

	**N = 17**
Year patient initiated on the line of therapy identified as T315I line of interest[1]	
Before 2010	1 (5.9)
2010–2014	9 (52.9)
2015–2019	7 (41.2)
Duration of the line of therapy identified as T315I line of interest[1,2], months	18.5 ± 20.6 [11.3]
Type of therapy received in the line of therapy identified as T315I line of interest[1]	
Ponatinib	10 (58.8)
Duration, months	28.7 ± 23.4 [24.0]
Dasatinib	3 (17.6)
Duration, months	8.3 ± 6.8 [11.3]
Allo-SCT	2 (11.8)
Other[3]	2 (11.8)
Duration, months	2.9 ± 0.0 [2.9]
Patients still on line of therapy identified as T315I line of interest[1,4] as of data collection date	6 (35.3)
Reasons for discontinuation[4,5]	11 (64.7)
Adverse events or intolerance[6]	9 (81.8)
Resistance	4 (36.4)
Signs of ineffectiveness	2 (18.2)
Suboptimal response	1 (9.1)
Progression to accelerated phase or blast phase	1 (9.1)
Other reasons	1 (9.1)
Acquired mutations[7]	1 (9.1)

Data are shown as mean ± standard deviation [median] or n (%).

Abbreviations: Allo-SCT, allogeneic stem cell transplantation; SD, standard deviation.

Notes:

[1] Line of therapy initiated after identification of T315I mutation.

[2] Calculated for 11 patients for whom treatment duration was reported.

[3] Other type of therapy included asciminib.

[4] Values were calculated based on all patients (including the two patients who received allo-SCT).

[5] Patients may have had more than one reason for treatment switch between lines of therapy; therefore, the sum may exceed 100%.

[6] Included adverse events and drug-drug interactions.

[7] Acquired mutation included T315I

Supplemental Table S4. Adverse events during third-line treatment for chronic myeloid leukemia

	3L+ cohort N = 157	T315I cohort N = 17
**AEs**[1]		
Numbers of AEs reported, mean ± SD [median]	2.7 ± 2.4 [2.0]	2.4 ± 2.9 [1.0]
Proportion of patients with AEs, n (%)	139 (88.5)	14 (82.4)
Infections	28 (17.8)	2 (11.8)
Asthenia	21 (13.4)	2 (11.8)
Abdominal pain	20 (12.7)	1 (5.9)
Peripheral arterial disease	17 (10.8)	2 (11.8)
Diarrhea (severe)	16 (10.2)	1 (5.9)
Liver toxicity	16 (10.2)	1 (5.9)
Muscle cramps	16 (10.2)	−
Pleural effusion	15 (9.6)	1 (5.9)
Arthralgia	14 (8.9)	2 (11.8)
Hypertension	14 (8.9)	1 (5.9)
Thrombocytopenia	14 (8.9)	3 (17.6)
Anemia	12 (7.6)	1 (5.9)
Musculoskeletal pain	12 (7.6)	2 (11.8)
Myalgia	12 (7.6)	2 (11.8)
Constipation	11 (7.0)	1 (5.9)
Dyslipidemia / cholesterol elevation	11 (7.0)	2 (11.8)
Oedema	11 (7.0)	1 (5.9)
Nausea	9 (5.7)	1 (5.9)
Dyspnea	8 (5.1)	2 (11.8)
Neutropenia	6 (3.8)	−
Rash (requiring more than topical therapy)	4 (2.5)	−
Fatigue	5 (3.2)	−
Headache	5 (3.2)	−
Pruritus	5 (3.2)	−
Pyrexia	5 (3.2)	−
Cough	4 (2.5)	−
Diabetes / hyperglycemia	4 (2.5)	−
Ischemic cerebrovascular events	4 (2.5)	2 (11.8)
Ischemic heart disease	4 (2.5)	1 (5.9)
Leukopenia	4 (2.5)	−
Vomiting	4 (2.5)	−
Back pain	3 (1.9)	−
Pulmonary arterial hypertension	2 (1.3)	−
Fluid retention	1 (0.6)	1 (5.9)
Nasopharyngitis	1 (0.6)	−
Pancreatitis	1 (0.6)	−
Myelosuppression	−	1 (5.9)
Pericardial effusion	−	1 (5.9)
Other[2]	88 (56.1)	7 (41.2)
None	14 (8.9)	2 (11.8)
Not assessed	0 (0.0)	0 (0.0)
Unknown / not sure	4 (2.5)	1 (5.9)

Data are shown as mean ± standard deviation [median] or n (%).

Abbreviations: AE, adverse event; Allo-SCT, allogeneic stem cell transplantation; NE, not evaluable; GvHD, graft vs. host disease.

Notes:

[1] Patients may have had more than one adverse event; therefore, the sum may exceed 100%.

[2] Other adverse events included acute myeloid leukemia, alopecia, amylasemia, angina, anorexia, aphasia, arrhythmia, arteriopathy, asthma, autoimmune and iatrogenic liver disease, Bell's palsy, bezoar, bilateral

cataract operated, bone tearing, breast cytosteatonecrosis nodule exeresis, bronchitis, c protein deficiency, capsular tendon rupture, cardiac decompensation, chest tightness, cholestasis, cognitive disorders, conjunctival icterus, conjunctivitis, cytolytic hepatitis, daily chills, depression, digestive disorders, digestive intolerance, diplopia, dizziness, dry skin, dysphonia, oedema, epigastralgia, erectile disorders, erythema, falls, gastric pain, general impairment, Gilbert syndrome, glomerulosclerosis, gout, gynecomastia, Hashimoto thyroiditis, heart failure, hematuria, hemiparesis, hemorrhoid, hot flushes, hydronephrosis, hyperthyroidism, hypertrophic heart disease, intraepithelial carcinoma excision, lack of appetite, leukocytosis, lipasemia, lumbar canal stenosis, lumbar radicular surgery, malignant tumor of the head of the pancreas, massive alcohol and tobacco poisoning, mastectomy for ductal carcinoma in situ, mnesic disorders, moderate undernutrition, myelodysplastic syndrome, neuropathy, night sweats, non-Hodgkin lymphoma, oligoarthritis, pneumonia, pneumopathy, polyuria crisis, prostatic adenocarcinoma, psychological disorders, pulmonary embolism, pyocyanic pneumonia, pyrosis, renal artery stenosis, renal colic, renal failure, respiratory failure, rhabdomyolysis, rhinitis, sciatica, severe mouth pains, sinusitis, skin lesion, sleep troubles, stents, tendonitis, tenosynovitis, thrombocytosis, thrombosis, thyroid cancer, tinnitus, total hip replacement, visual disorders, weight gain, and xanthelasma..

Supplemental Figure S1. Study design scheme. Abbreviations: 3L, third line; CML-CP, chronic myeloid leukemia in chronic phase.

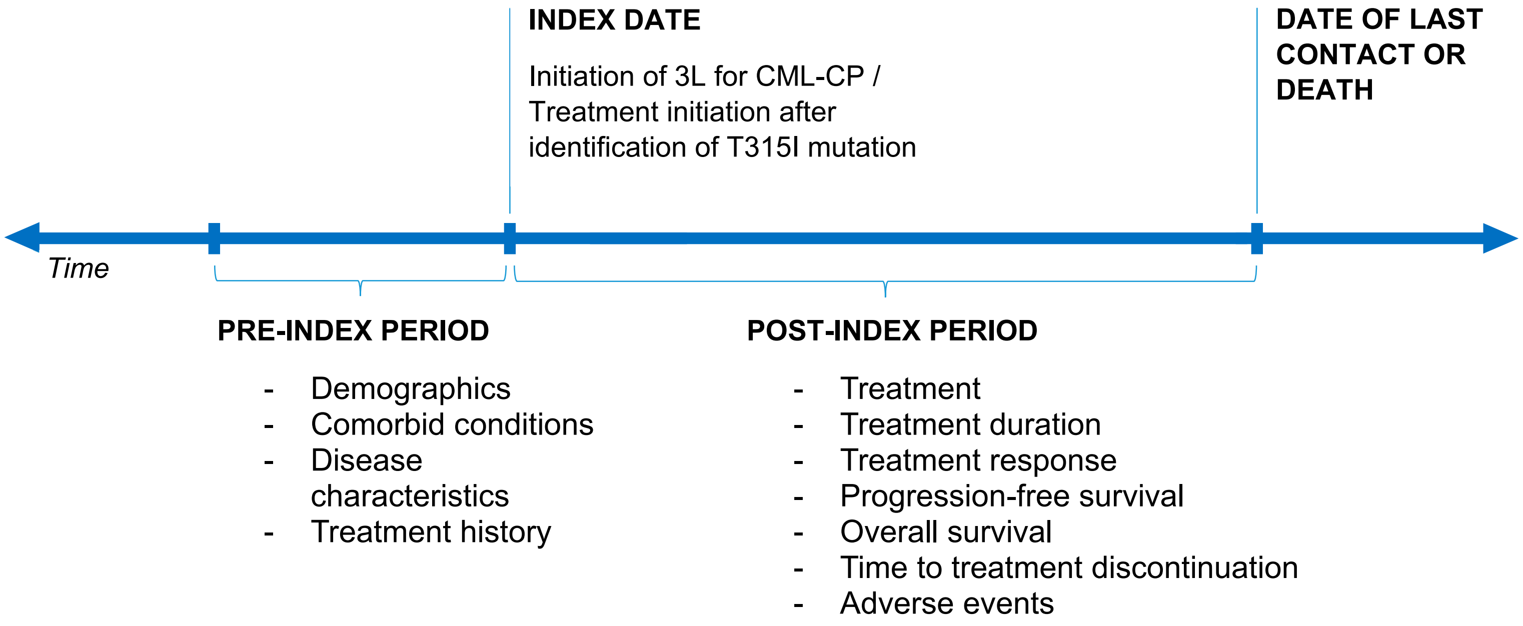

Supplement: Supplementary file 1 [file cancers-15-04161-s001.zip › cancers-2522615-supplementary.pdf]
